# Dysarthria in hereditary spastic paraplegia type 4

**DOI:** 10.1016/j.clinsp.2022.100128

**Published:** 2022-12-05

**Authors:** Lais Alves Jacinto-Scudeiro, Rui Rothe-Neves, Vanessa Brzoskowski dos Santos, Gustavo Dariva Machado, Daniela Burguêz, Marina Martins Pereira Padovani, Annelise Ayres, Rafaela Soares Rech, Carelis González-Salazar, Marcondes Cavalcante França Junior, Jonas Alex Morales Saute, Maira Rozenfeld Olchik

**Affiliations:** a Postgraduate Program in Medicine, Medical Sciences, Universidade Federal do Rio Grande do Sul, Porto Alegre, RS, Brazil; b Phonetics Laboratory of the Faculty of Letters, Universidade Federal de Minas Gerais, Belo Horizonte, MG, Brazil; c Medical Genetics Service, Hospital de Clínicas de Porto Alegre, Porto Alegre, RS, Brazil; d Faculdade de Ciências Médicas da Santa Casa de São Paulo, São Paulo, SP, Brazil; e Universidade Federal de Ciências da Saúde de Porto Alegre, Porto Alegre, RS, Brazil; f Postgraduate Program in Medical Pathophysiology, Universidade Estadual de Campinas, São Paulo, SP, Brazil; g Department of Neurology, Universidade Estadual de Campinas, São Paulo, SP, Brazil; h Internal Medicine Department, Faculdade de Medicina Universidade Federal do Rio Grande do Sul, Porto Alegre, RS, Brazil; i Department of Surgery and Orthopedics, Faculdade de Odontologia, Universidade Federal do Rio Grande do Sul, Porto Alegre, RS, Brazil

**Keywords:** Spastic Paraplegia, Hereditary, Speech, Dysarthria, Speech Disorders

## Abstract

**Objective:**

To describe the speech pattern of patients with hereditary Spastic Paraplegia type 4 (SPG4) and correlated it with their clinical data.

**Methods:**

Cross-sectional study was carried out in two university hospitals in Brazil. Two groups participated in the study: the case group (*n* = 28) with a confirmed genetic diagnosis for SPG4 and a control group (*n* = 17) matched for sex and age. The speech assessment of both groups included: speech task recording, acoustic analysis, and auditory-perceptual analysis. In addition, disease severity was assessed with the Spastic Paraplegia Rating Scale (SPRS).

**Results:**

In the auditory-perceptual analysis, 53.5% (*n* = 15) of individuals with SPG4 were dysarthric, with mild to moderate changes in the subsystems of phonation and articulation. On acoustic analysis, SPG4 subjects’ performances were worse in measurements related to breathing (maximum phonation time) and articulation (speech rate, articulation rate). The articulation variables (speech rate, articulation rate) are related to the age of onset of the first motor symptom.

**Conclusion:**

Dysarthria in SPG4 is frequent and mild, and it did not evolve in conjunction with more advanced motor diseases. This data suggest that diagnosed patients should be screened and referred for speech therapy evaluation and those pathophysiological mechanisms of speech involvement may differ from the length-dependent degeneration of the corticospinal tract.

## Highlights

• Prevalence of dysarthria in SPG4 is greater than previously considered.
• The dysarthria in SPG4 is mild or moderate.
• The subsystems of speech more affected are articulatory and phonation.

## Introduction

Hereditary Spastic Paraplegias (HSP) are a heterogeneous group of genetic diseases characterized by progressive lower limb spasticity.^1^^,^^2^ HSP are usually classified on clinical grounds into pure or complex forms. Only pyramidal syndrome is found in pure forms (changes in vibratory sensitivity and neurogenic bladder are also accepted), whereas in complex forms, the pyramidal syndrome is accompanied by dysfunction in other neurological systems or by systemic involvement (eg: ataxia, epileptic seizures, cognitive decline, dementia, amyotrophy, extrapyramidal signs, peripheral neuropathy, and deafness; visual abnormalities; skin changes, among others).^3^ There are 83 Spastic Paraplegia (SPG) *loci/*genes known to date^4^ and this study will focus on Spastic Paraplegia type 4 (SPG4).

SPG4 is the most frequent subtype of HSP, representing between 37% and 60% of cases with an autosomal dominant inheritance in Europe and Brazil and can be found in up to 10%‒15% of isolated cases with a pure presentation. The age of onset of SPG4 is very variable and can occur from early childhood to the eighth decade of life. Despite being described as a pure form of HSP, rare cases may present complicating features.^5^, ^6^, ^7^, ^8^

Dysarthria is a disturbance in the control of speech mechanisms due to damage in the central or peripheral nervous system that affects the communication of patients with several neurological disorders. It reflects abnormalities in subsystems of speech: breathing, phonation, articulation, resonance, and/or prosody, due to irregularities in strength, speed, amplitude, endurance, tone, or precision of the speech mechanism, impairing intelligibility.^9^^,^^10^ The use of auditory perceptual and acoustic speech assessment has been studied as a way to better understand neurological illnesses and even as a diagnostic resource.^11^^,^^12^ Studies with other neurogenetic conditions such as Huntington's disease and ataxias have shown abnormalities in the acoustic analysis of speech even in preclinical stages of these diseases, highlighting its potential as a disease biomarker.^13^, ^14^, ^15^, ^16^, ^17^

Although dysarthria has been described in the case series of SPG4 patients^7^^,^^18^, ^19^, ^20^ as a negligible complaint, these previous studies performed a subjective assessment of speech (through a questionnaire) by physicians, without a completed speech assessment (acoustic and auditory perceptual analysis).

Therefore, the present study aimed to perform a detailed characterization of the main aspects of speech in subjects with SPG4, which can contribute to a better understanding of speech abnormalities in this disease, and generate relevant information that can assist in improving the care and quality of life of these individuals.

## Methods

### Design and subjects

This is an exploratory, cross-sectional study conducted at two university hospitals in the Brazilian cities Porto Alegre and Campinas. The study included twenty-eight consecutive patients with a genetically confirmed diagnosis of SPG4 followed at the Neurogenetics outpatient clinics of these hospitals, from December 2016 to August 2018.

Seventeen healthy control individuals matched by sex and age were recruited from the local community in Porto Alegre, Brazil. In order to rule out the presence of any disease that could interfere with the speech in the control group, all individuals answered questions about diagnoses, surgeries, and medication use. The exclusion criteria for both groups were individuals without Brazilian Portuguese as a native language, history of other previous neurological events, sensory or motor disorders, systemic diseases and/or structural changes that affected the voice and/or speech.

### Ethical compliance statement

The project was approved by the Ethics Committee of Hospital de Clínicas de Porto Alegre da Universidade Federal do Rio Grande do Sul under review number 2017‒0012, which follows the Declaration of Helsinki. Informed written consent was obtained from all individuals’ prior participation.

### Procedures

Individuals with SPG4 underwent the speech assessments and answered the following questionnaires:

Socio-demographic questionnaire: structured questionnaire containing questions regarding general patient data, such as age; sex; schooling.

Neurological Evaluation: neurological severity was assessed by the Brazilian Portuguese version of the Spastic Paraplegia Rating Scale (SPRS, range: 0–52, crescent in severity).^21^ Disease duration and age of onset of the first motor symptom were reported by patients and their relatives.

The speech assessment was performed in both groups with speech collection, auditory-perceptual and acoustic analysis, described below:

Speech collection: Speech samples were recorded in an acoustically treated environment using the software Audacity. A KARSECT HT-9 microphone with the Andrea Pureaudio USB adapter was positioned approximately 5 cm from the subject's lips. The assessment includes the tasks to test the five subsystems of speech: phonation (sustaining the vowel /a/ in a single breath), resonance (alternation of the vowel sequence [i] and [u], repeatedly in a single breath­), respiration (sustaining the vowel /a/ in a single breath), and articulation (alternating the sequence of syllables [pataka] as fast as personal capacity allowed, repeatedly in a single breath named as Diadochokinesis (DDK).

Auditory-perceptual analysis: The analysis was carried out by 5 speech therapists trained and with a coefficient of agreement Kappa index ≥ 0.90, blinded to diagnosis. All speech therapists had at least three years of experience in speech analysis. Before the speech analysis procedures, different types of speech alterations were presented and evaluated for auditory training. The examiners heard the speech samples in random order and analyzed the subsystems of speech (phonation, articulation, breathing, resonance and prosody) based on the dimensions described by Duffy.^9^ The authors used a severity classification on a 0 to 4 scale of abnormality (0 = normal, 1 = mild dysarthria, 2 = moderate dysarthria, or 3 = severe dysarthria).

Acoustic analysis: The authors used a script^22^, ^23^, ^24^ to detect syllable nuclei from the intensity peaks and measure speech rate automatically with Praat.^25^ The parameters analyzed were based on Rusz et al.^26^ and Vogel and Maruff.^27^ Speech variables were Phonation (Jitter (rap), Shimmer (local), Fundamental Frequency (f0), Fundamental Frequency DP, Harmonics-to-Noise Ratio ‒ HNR), Articulation (Number of syllables, Number of pauses, Duration, Phonation time, Phonation rate, Speech rate, Articulation rate, Average Syllable Duration ‒ ASD), Breathing (Maximum Phonation Time ‒ MPT), Resonance (2^nd^ vowel Formant (F_2_) [i] and [u]).

### Data analysis

Descriptive data analysis was performed using absolute and relative frequencies, mean and standard deviation, or median and interquartile range. The statistical tests were selected according to the distribution of data provided by the Shapiro-Wilk test and histograms.

Comparisons between SPG4 and control subjects were performed using Student's *t*-test. The 95% Confidence Interval (95% CI) for differences in means between groups was also provided. For the analysis of categorical variables, Fisher's exact test was used. Statistical significance was defined as p < 0.05.

## Results

A total of 45 individuals were analyzed, 28 from the case group and 17 from the Control Group (CG). The SPG4 group had a mean disease duration of 28.96 (± 15.95) years and a mean score of 19.61 (± 10.40) on the neurological severity scale. No significant differences were found between groups for age (*p* = 0.747) and sex (0.604). The sociodemographic data of both groups are shown in Table 1.

**Table 1:** Sociodemographic data of both groups.

Variable	Control group (*n* = 17)	Case group (*n* = 28)	*p*-value
Age	51.47 ± 11.60	50.32 ± 1.32	0.747
Sex female	52.9% (9)	53.6% (15)	0.604
Male	47.1% (8)	46.4% (13)	
Disease time	‒	28.96 ± 15.95	‒
SPRS	‒	19.61 ± 10.40	‒
Age of onset	‒	31.25 ± 16.99	‒

SPG, Hereditary Spastic Paraplegia type 4; SPRS, Spastic Paraplegia Rating Scale.

Table 2 presents the auditory perceptual analysis of the groups in each of the five subsystems of speech. In the SPG4 group, 53.5% (*n* = 15) of the individuals presented dysarthria with mild to moderate alterations in the subsystem's phonation, breathing, resonance and articulation. A statistically significant difference was observed between the groups in subsystems phonation (*p* = 0.034) and in articulation (*p* = 0.004).

**Table 2:** Analysis of the auditory-perceptual analysis.

Variable	Control group	Case group	*p*-value
Breathing			
Normal	15 (88.2%)	25 (89.3%)	0.650
Mild impairment	2 (11.8%)	2 (7.1%)	
Moderate impairment	0 (0.0%)	1 (3.6%)	
Phonation			
Normal	14 (82.4%)	13 (46.4%)	0.034
Mild dysarthria	3 (17.6%)	9 (32.1%)	
Moderate impairment	0 (0.0%)	6 (21.4%)	
Resonance			
Normal	16 (94.1%)	24 (85.7%)	0.616
Mild impairment	1 (5.9%)	3 (10.7%)	
Moderate impairment	0 (0.0%)	1 (3.6%)	
Articulation			
Normal	17 (100.0%)	15 (53.6%)	0.004
Mild impairment	0 (0.0%)	9 (32.1%)	
Moderate impairment	0 (0.0%)	4 (14.3%)	
Prosody			
Normal	17 (100.0%)	28 (100.0%)	-
Mild impairment	0 (0.0%)	0 (0.0%)	
Moderate impairment	0 (0.0%)	0 (0.0%)	
Severity classification			
Normal	15 (88.2%)	13 (46.4%)	0.017
Mild dysarthria	3 (17.6%)	9 (32.1%)	
Moderate dysarthria	0 (0.0%)	6 (21.4%)	

In the acoustic analysis, the authors verified that the SPG4 subjects’ performances were worse in measurements related to breathing (maximum phonation time), articulation (number of syllables, phonation time, speech rate, articulation rate e ASD) e resonance (F_2_[i] and F_2_[u]). There was no statistically significant difference between the groups in the phonation variables. These data are presented in Table 3.

**Table 3:** Descriptive analysis of the studied sample stratified by group.

Task	Variable	Control group	Case group	Difference (95%)	*p*-value
Sustained vowel /a/	Jitter rap	0.24 ± 0.14	0.38 ± 0.35	-0.14 (-0.29‒0.01)	0.077
	Shimmer local	7.58 ± 4.00	8.98 ± 4.21	-1.4 (-3.97‒1.16)	0.277
	F_0_ mean	164.03 ± 45.50	165.61 ± 49.12	-1.58 (-31.22‒28.0)	0.915
	F_0_ standar desviation	18.91 ± 24.88	41.10 ± 61.51	-22.19 (-48.69‒4.30)	0.098
	MPT	8.97 ± 6.19	5.41 ± 4.87	3.56 (0.21‒6.91)	0.038
	HNR	15.25 ± 3.97	14.21 ± 3.89	1.04 (-1.39‒3.47)	0.393
DDK	Number of syllables	27.65 ± 15.70	16.11 ± 5.80	11.54 (3.23‒19.84)	0.009
	Number of pause	0.35 ± 1.22	0.79 ± 1.28	-0.35 (-1.21‒0.35)	0.271
	Duration (s)	5.30 ± 3.89	3.89 ± 1.04	1.41 (-0.63‒3.43)	0.164
	Phonation time (s)	4.84 ± 3.89	3.27 ± 0.97	1.57 (-0.63‒3.43)	0.127
	Speech rate	5.56 ± 1.05	4.19 ± 1.09	1.36 (0.70‒2.03)	<0.001
	Articulation rate	6.13 ± 0.92	4.94 ± 0,94	1.19 (0.60‒1.77)	<0.001
	ASD	0.16 ± 0.02	0.21 ± 0.04	-0.04 (-0.06‒0.02)	<0.001
Alternation the vowel sequence I-U	F_2_ [i]	2410.29 ± 259.07	2377.51 ± 343.85	32.78 (-162.53‒228.09)	0.737
	F_2_ [u]	1026.35 ± 323.15	786.90 ± 148.92	239.44 (65.84‒413.05)	0.009
	F_2_ [i]/ F_2_ [u]	2.58 ± 0.71	3.11 ± 0.67	-0.52 (-0.94–0.09)	0.018

DDK, Diadochokinesis; MPT, Maximum Phonation Time; HNR, Harmonics-to-Noise Ratio; Phonation Time, Proportion of speaking time on total time; Speech Rate, Syllables per second; Articulation rate, Syllables per second (excluding pauses); ASD, Average Syllable Duration; F_2_, Frequency of the second formant; F_2_ [i]/ F_2_ [u], Frequency of the second formant in [i] divided by the frequency of the second formant in [u]; (s), Seconds.

No statistically significant correlations were found between acoustic variables and clinical disease data (time of disease and neurological severity). There was a significant correlation between the variables of the breathing, resonance, and articulation subsystems with the age of onset. These data can be found in Table 4.

**Table 4:** Correlation between clinical variables and acoustic analysis.

Variable	SPRS		Disease time		Age of onset	
	*R*	*p*-value	*R*	*p*-value	*R*	*p*-value
Jitter rap	0.147	0.456	0.147	0.456	-0.225	0.138
Shimmer local	0.093	0.639	0.291	0.133	-0.166	0.275
F_0_ mean	0.214	0.273	0.045	0.821	-0.024	0.878
F_0_ standard deviation	-0.069	0.729	0.125	0.527	-0.217	0.152
MPT	-0.023	0.908	-0.191	0.329	0.308	0.039
HNR	-0.012	0.952	-0.279	0.150	0.126	0.410
Number of syllables	0.173	0.379	0.057	0.774	0.474	0.001
Number of pause	-0.164	0.404	-0.013	0.948	-0.162	0.287
Duration (s)	-0.226	0.247	0.006	0.976	0.269	0.074
Phonation time (s)	-0.238	0.223	-0.046	0.816	0.294	0.050
Speech rate	0.090	0.650	0.144	0.465	0.528	<0.001
Articulation rate	0.036	0.857	0.190	0.333	0.529	<0.001
ASD	-0.139	0.482	-0.292	0.132	-0.501	<0.001
F_2_ [i]	0.283	0.145	0.036	0.855	0.049	0.750
F_2_ [u]	-0.73	0.714	-0.316	0.101	0.453	0.002
F_2_ [i]/ F_2_ [u]	0.220	0.260	0.195	0.319	-0.347	0.20

* Pearson correlation.

R, Pearson correlation coefficient; SPRS, the Spastic Paraplegia Rating Scale; MPT, Maximum Phonation Time; HNR, Harmonics-to-Noise Ratio; Phonation Time, Proportion of speaking time on total time; Speech Rate, Syllables per second; Articulation rate, Syllables per second (excluding pauses); ASD, Average Syllable Duration; F_2_ , Frequency of the second formant; F_2_ [i]/ F_2_ [u], Frequency of the second formant in [i] divided by the frequency of the second formant in [u]; (s), Seconds.

## Discussion

In the present study, the authors have found a greater than previously reported prevalence of speech abnormalities in SPG4 on speech-language pathologists’ assessments. Auditory Perceptual Analysis (APA), with 53.5% (*n* = 15) of individuals with SPG4 being dysarthric. Regarding the subsystems of speech, 53.5% of patients presented phonation and 46.4% presented articulation impairments. Acoustic analysis amplified speech alterations in SPG4, portraying subclinical breathing and resonance of speech impairments. When the authors correlated the disease duration and the motor scale, no significant correlations were found. Thus, the data suggest that speech disorders do not evolve in conjunction with a more advanced motor disease and that the pathophysiological basis of speech involved in the disease is somehow distinct from duration-dependent involvement of the corticospinal tract.

### Detailed characterization of speech abnormities in SPG4

#### Greater prevalence of speech abnormalities than previously considered

More than half of the evaluated SPG4 patients presented abnormal articulation or phonation on auditory-perceptual analysis of speech, with most abnormalities classified as mild. Some studies have shown the presence of dysarthria in this population, but with a very low prevalence, varying from 3 to 5%.^7^^,^^20^ It is observed that dysarthria in these studies was evaluated by the neurologist's clinical impression, without speech assessment and without acoustic and/or auditory perceptual analysis, suggesting that this previous prevalence would probably be underestimated. Thus, the present data indicate that dysarthria diagnosis assessed by speech-language pathologists is more sensitive than previously reported by self-reports and by the impression of other professionals,^7^^,^^20^ being an underdiagnosed symptom of the disease.

### Acoustic analysis revealed additional speech abnormalities in SPG4

Acoustic analysis of speech found impairments in the subsystems: articulation, breathing and resonance. Complementing the acoustic analysis, the auditory perceptual analysis also found changes in the subsystem phonation. For articulation assessment, the authors used the DDK and alternation vowel sequence task. DDK tests the ability to perform fast repetitions of relatively simple patterns of opposing muscle contractions, providing information on motor control and integration. The alternation vowel sequence task is the ability of rapid and rhythmic transition of tongue articulators and assesses articulatory mobility. DDK task is one of the most used tests to evaluate the integrity of the oral motor system in neurological diseases.^28^ In patients with cerebellar ataxia, chorea, or Parkinson's disease, there is a decrease in the speed and quality of movements of oral DDK.^29^, ^30^, ^31^ When analyzing the subsystems of phonation, the authors investigate the vocal quality, frequency, and intensity of produced sounds and the stability of sound emission. SPG4 patients presented rough and breathy voices, and one possibility is due to the presence of muscle tension and stiffness due to the disease, leading to irregularities in vocal fold vibration (rough voice) and difficulties in completely closing vocal folds (breathy voices).

The subsystem breathing was altered in SPG4 patients in the acoustic analysis. Maximum phonation time indicates the subject's ability to control the aerodynamic forces of pulmonary airflow and the myoelastic forces of the larynx and it holds as a measure of coordination between breathing and phonation.^32^ In the present study, patients with SPG4 had lower maximum phonation time, which might result from the lack of pneumo-phono-articulatory coordination due to the disease, resulting in insufficient airflow and marked speech unintelligibility.^9^^,^^32^ Remarkably, alterations in maximum phonation time were also seen in other movement disorders, such as Multiple System Atrophy and Parkinson´s Disease.^11^

Acoustics and auditory-perceptual analysis agreed with changes in the articulation. In the subsystem breathing, it is noted that auditory-perceptual analysis is based on speech functionality, identifying aspects of pneumo-phono-articulatory incoordination (identified in phonation), while acoustics is sensitive and manages to separate changes in the subsystem breathing and phonation. The ratio between the second formant of the vowel [i] and the second formant of the vowel [u] is a measure of vowel formant centralization, which indicates a reduced range of articulatory movements. In this case, the authors measured the second Formant (F_2_), which varies with anterior-posterior movements of the tongue and rounding and ungrounding of the lips. The higher the value of the ratio between the second formant of the vowel [i] and the second formant of the vowel [u], the greater the articulatory movement (lips and tongue). The limitation in the forward and backward movement of the tongue in the oral cavity results in a decreased proportion of the formant ratio; a hypothetical case with [i] and [u] produced at the same central pivot point would result in a ratio of 1. Based on published data, the authors estimate F_2_[i]/F_2_[u] around 3.29 for women and 2.88 for men,^33^ but as a ratio, it seems to have no gender effects. In SPG4 the authors did not find evidence that the case group differs from the controls as to resonance.

Finally, it is important to emphasize that acoustic analysis has become an essential tool for understanding speech patterns in the studied population, complementing the auditory perceptual analysis and showing subclinical and inaudible changes in some motor bases of speech. These findings show that there are variables that can be potential biomarkers for speech disorders in SPG4, so the authors suggest the development of a minimal protocol for acoustic analysis in this population with measures of maximum phonation time and speech rate, and articulation rate (DDK).

### Study limitations

The study limitations were due to the exploratory nature of the study, the authors neither performed sample size calculation nor defined the study power and main outcome. In addition, as it is a cross-sectional study, it was not possible to verify whether the evolution of speech goes along with the course of the disease, longitudinal investigation is needed.

## Conclusion

Dysarthria found in SPG4 patients was mild and related to impairments in articulatory and phonation subsystems, and also in coordination between the phonation and breathing subsystems. Auditory perceptual analysis corroborated the findings of the acoustic analysis, which also showed subclinical abnormalities. Apparently, speech impairments do not develop in conjunction with advancing motor symptoms, suggesting that diagnosed patients should be screened and referred for speech therapy evaluation during the disease course. Apparently, the pathophysiological basis of speech involvement in the disease is somehow distinct from the length-dependent involvement of the corticospinal tract.

## Disclosures

The National Council for Scientific and Technological Development – CNPq (460941/2014-3) and FIPE-HCPA (GPPG-HCPA 17-0012) granted this study. Coordenação de Aperfeiçoamento de Pessoal de Nível Superior ‒ Brasil (CAPES) ‒ Finance Code 001 supported MPB and LAJS. CNPq supported RRN (316036/2021-8) and DB, and the Fundação de Amparo à Pesquisa do Estado do Rio Grande do Sul (FAPERGS) supported GDM. Public Brazilian agencies were neither involved in the study design and protocol, data collection, analysis, interpretation, writing the report, nor deciding to submit the paper for publication. All authors report no conflict of interest related to the study.

## CRediT authorship contribution statement

**Lais Alves Jacinto-Scudeiro:** Conceptualization, Data curation, Formal analysis, Visualization, Writing – original draft, Writing – review & editing. **Rui Rothe-Neves:** Formal analysis, Visualization, Writing – original draft, Writing – review & editing. **Vanessa Brzoskowski dos Santos:** Data curation, Writing – review & editing. **Gustavo Dariva Machado:** Data curation, Writing – review & editing. **Daniela Burguêz:** Data curation, Writing – review & editing. **Marina Martins Pereira Padovani:** Conceptualization, Writing – original draft, Writing – review & editing. **Annelise Ayres:** Data curation, Writing – review & editing. **Rafaela Soares Rech:** Formal analysis, Visualization, Writing – review & editing. **Carelis González-Salazar:** Data curation, Writing – review & editing. **Marcondes Cavalcante França Junior:** Data curation, Writing – review & editing. **Jonas Alex Morales Saute:** Conceptualization, Data curation, Formal analysis, Visualization, Writing – original draft, Writing – review & editing. **Maira Rozenfeld Olchik:** Conceptualization, Writing – original draft, Writing – review & editing.

## Declaration of Competing Interest

The authors declare no conflicts of interest.
